# A framework for identifying opportunities for multisectoral action for drowning prevention in health and sustainable development agendas: a multimethod approach

**DOI:** 10.1136/bmjgh-2024-016125

**Published:** 2024-08-22

**Authors:** Justin-Paul Scarr, David R Meddings, Caroline Lukaszyk, Joanne Adrienne Vincenten, Aminur Rahman, Steve Wills, Jagnoor Jagnoor

**Affiliations:** 1 The George Institute for Global Health, University of New South Wales, Sydney, NSW, Australia; 2 Royal Life Saving Society - Australia, Sydney, NSW, Australia; 3 Department of the Social Determinants of Health, Division of UHC/Healthier Populations, World Health Organization, Geneva, Switzerland; 4 Health, UNICEF, New York, NY, USA; 5 Centre for Injury Prevention and Research, Dhaka, Bangladesh; 6 Royal National Lifeboat Institution, Poole, UK

**Keywords:** Injury, Health policy

## Abstract

**Introduction:**

The 2023 World Health Assembly resolution 76.18 committed the World Health Organization to the coordination of drowning prevention efforts, including those of United Nations (UN) agencies. Here, we aim to map drowning prevention linkages across UN Agency agendas, make recommendations to guide global strategies and inform the development of the Global Alliance and a Global Strategy for drowning prevention.

**Methods:**

We applied a qualitative multimethod approach, including document review, key informant interviews, an interagency workshop and international conference panel discussion, to refine data and create our recommendations. We developed a framework to identify intersections between health and sustainable development agendas and applied it to map intersections and opportunities for the integration of drowning prevention across relevant UN Agency agendas.

**Results:**

Our framework categorised intersections for drowning prevention in UN Agendas according to potential for (a) shared understandings of problems and solutions, (b) shared capacities, guidelines and resources and (c) shared governance and strategic pathways, noting that some factors overlap. We present our Position, Add, Reach and Reframe approach to outlining opportunities for the integration of drowning prevention in health and sustainable development agendas. Our results emphasise the importance of establishing approaches to the Global Alliance and Global Strategy that ensure high-level political advocacy is converted into solutions for affected communities. We recommend using research to inform effective action, building capacity and best practices, and promoting evaluation frameworks to incentivise and verify progress.

**Conclusion:**

Our study identifies opportunities to expand drowning prevention efforts and to build Member State capacity to reduce drowning risk through evidence-informed measures that address vulnerabilities, exposures, hazards and build population-level resilience to drowning. Our framework for identifying opportunities for integration of drowning prevention across a multisectoral set of agendas offers a research and policy toolkit that may prove useful for other policy areas.

**WHAT IS ALREADY KNOWN ON THIS TOPIC**

- Previous studies identify strategic priorities for drowning prevention across domains including engagement with health and sustainable development agendas, high-level political advocacy and the strengthening of inclusive global leadership and governance.
- Resolutions at the United Nations General Assembly and World Health Assembly reinforce the need for coordination of UN agencies efforts and expansion of multisectoral approaches to drowning prevention.

**WHAT THIS STUDY ADDS**

- Our study identifies intersections for drowning prevention in UN Agency agendas through the development and application of a framework focused on identifying potential for (a) shared understandings of problems and solutions, (b) shared capacities, guidelines and resources and (c) shared governance and strategic pathways.
- We identify opportunities for integrating drowning prevention within other agendas by applying the Position, Add, Reach and Reframe approach to strengthen multisectoral action for drowning prevention.
- Our findings provide recommendations and a logic model to guide the development and implementation of the Global Alliance and Global Strategy for Drowning Prevention.

**HOW THIS STUDY MIGHT AFFECT RESEARCH, PRACTICE OR POLICY**

- Our framework could be applied to assess opportunities for drowning prevention in child and adolescent health, climate health and resilience, and disaster risk reduction agendas, and may provide a research tool for use in other policy areas.
- Our approach to integrating drowning prevention could be applied to establish cobenefits and opportunities in multisectoral partnerships at regional, national and community level.

## Introduction

 Global drowning mortality exceeds 236 000 deaths annually and drowning is a leading killer of children, adolescents and young adults.^1^ Vulnerabilities and exposures to drowning vary across populations and contexts,^2^ and as such drowning prevention cuts across a wide range of stakeholders and sectors. Despite long- term advocacy and the availability of effective evidence-informed interventions,^3^ drowning is generally overlooked in public health and sustainable development policy.^4^ Recent traction on high-level political agendas aspires to turn that tide.^3^

In April 2021, the United Nations General Assembly (UNGA) resolution A/RES/75/273^5^ reinforced that drowning is preventable, that scalable interventions for drowning prevention exist, and that multisectoral coordination is needed for effective action.^5^ Then, in May 2023 the World Health Assembly (WHA) adopted resolution WHA76.18, which calls on the World Health Organization (WHO) to coordinate multisectoral drowning prevention efforts across United Nations (UN) Agencies, Member States, international development partners and civil society.^6^

Two subsequent commitments foreshadow WHO’s approach: (1) the formation of a Global Alliance for Drowning Prevention (Global Alliance) to strengthen global coordination and (2) development of a Global Strategy for Drowning Prevention (Global Strategy). In July 2023, WHO established the Global Alliance by initially inviting the International Maritime Organization (IMO), the Food and Agriculture Organization (FAO), UN Development Programme (UNDP), United Nations Children’s Fund (UNICEF) and five non-state actors (described elsewhere) to participate in the preliminary Global Alliance steering committee.

The need for increased global coordination and global strategies for drowning prevention was first proposed at the World Conference on Drowning Prevention (WCDP) in Vietnam in 2011,^7 8^ then recommended in the WHO’s Global Report on Drowning in 2014^4^ and has been explored in research since.^39^,^12^ Strategic priorities have been identified to build cohesion, strengthen global inclusive governance, increase accountability, drive multisectoral action and prompt country-level responses.^9 11^

This study aims to (1) build a framework for identifying intersections between health and sustainable development agendas, (2) apply the framework to map intersections for drowning prevention in UN Agency agendas, (3) identify opportunities for integration in UN Agency agendas and (4) identify themes and lessons to inform the development and implementation of the Global Alliance and Global Strategy for Drowning Prevention.

## Methods

**Figure 1:**
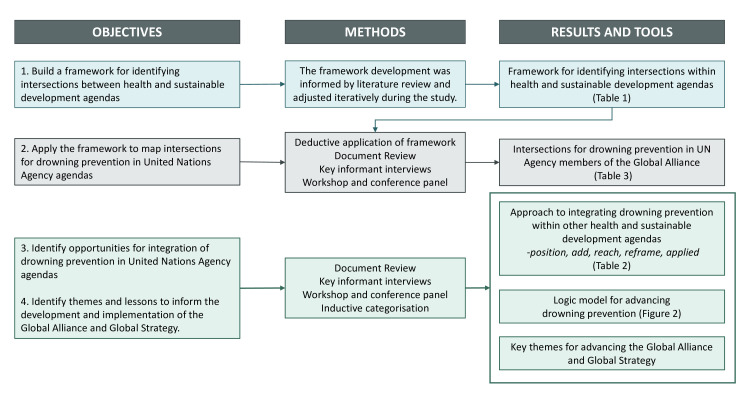
Study flow chart.

A multimethod approach drew data from (1) document review, (2) key informant interviews, (3) an interagency workshop and (4) an international conference panel discussion, all informed by the study advisory group (figure 1). This study is reported in accordance with the Consolidated criteria for Reporting Qualitative research.^13^

Throughout our methods, we apply the consensus-based definition where drowning prevention is defined as a multidisciplinary approach that reduces drowning risk and builds resilience by implementing evidence-informed measures that address hazards, exposures and vulnerabilities to protect an individual, community or population against fatal and non-fatal drowning.^2^

### Advisory group

Our study was guided by the Global Alliance steering committee which acted as study advisory group and reviewed study objectives, interim analysis, results and interpretation. The steering committee included representatives from UN agencies; WHO, IMO, FAO, UNDP, UNICEF and non-state actors; Bloomberg Philanthropies, Centre for Injury Prevention and Research, Bangladesh (CIPRB), Royal Life Saving Society—Australia (RLSSA), Royal National Lifeboat Institution (RNLI) UK and The George Institute for Global Health (TGI).

In addition to key informant interviews, Global Alliance steering committee members participated in an interagency workshop and international conference panel discussion, both held during the WCDP 2023 and reviewed data from each method.

### Building a framework for identifying intersections

In line with aim (1) and to develop the framework for identifying intersections between health and sustainable development agendas, we leveraged literature from our prior examination of global agenda setting^3^ and a scoping review exploring multisectoral action for drowning prevention.^10^

We considered terms ‘framework,’ and ‘mapping,’ ‘intersection,’ or ‘interaction’ with target areas of ‘health’ and ‘development agenda’. Further, we conducted a hand search of relevant cross-references from both grey and peer-reviewed literature. Framework development was informed by a literature review of approaches to the analysis of linkages between Sustainable Development Goals (SDGs),^14 15^ the integration of health across SDGs,^16^ reviewing governance in multisectoral collaborations^17^,^19^ and frameworks on the effectiveness of global health networks.^20 21^

### Applying the framework to map intersections and opportunities for integration

The draft framework was then applied to the following study aims (2) apply the framework to map intersections for drowning prevention in UN Agency agendas, along with document review, key informant interviews, interagency workshop and international conference panel discussion. The methods were then applied to (3) identify opportunities for integration in UN Agency agendas and (4) identify themes and lessons to inform the development and implementation of the Global Alliance and Global Strategy.

### Document review

We used document review,^22^ to identify intersections and opportunities for integration for drowning prevention in UN Agency agendas. The websites of the IMO, FAO, UNDP, UNICEF and UN library were searched for ‘drowning’ to identify potential alignment in objectives, priorities, key activities and governance of the UN Global Alliance members.^2^ We also reviewed global health partnerships where WHO plays a coordinating role to identify strategic approaches to leadership and governance (https://www.who.int/about/collaboration/partnerships) .

### Key informant interviews, interagency workshop and international conference panel discussion

Key informant interviews (eight) were conducted with all Global Alliance steering committee members, other than FAO representatives, who were not available. Key informants for this study were restricted to Global Alliance steering committee members for three reasons. First, the meetings of the Global Alliance provided opportunity and access to the key informants. Second, the initial UN Agency members provided a sample for consideration of intersections and integration of drowning prevention within health and sustainable development agendas. Finally, the Global Alliance membership is defined by WHO.

Key informant interviews were approximately 1 hour in duration, were conducted online throughout November 2023 and were recorded. Interviews were based on document review findings and questions adjusted for informant background. Interviews were transcribed using caption feature, coded according to the framework for identifying intersections in health and sustainable development agendas shown in table 1. The interview guide can be found in online supplement 1.

**Table 1:** Framework for identifying the intersections in health and sustainable development agendas

Domains	Factors	Objectives
Identifying and building on shared understandings of problems and solutions	Shared problems	To identify areas of the health and sustainable development agenda where identifiable mortality, morbidity, socioeconomics costs can be attributed to the target health issue.
	Affected groups	To identify populations addressed by the health and sustainable development agenda, which have commonality to affected groups for the target health issue.
	Workable solutions	To identify solutions (interventions) delivered by the health and sustainable development agenda that relate directly to those delivered in the target health issue.
Identifying and building on shared capacities, guidelines and resources	System capacities	To identify system-level capacities in the health and sustainable development agenda that may have applicability to the target health issue.
	Standards and guidelines	To identify standards or guidelines in the health and sustainable development agenda that may have applicability to the target health issue.
	Resources	To identify human and financial resources in the health and sustainable development agenda that may have applicability to the target health issue.
Identifying and building on shared governance pathways	Global institutions	To identify global mechanisms in the health and sustainable development agenda that may have applicability to the target health issue.
	Global strategies and plans	To identify global strategies or plans in the health and sustainable development agenda that may have applicability to the target health issue.
	Multistakeholder platforms	To identify the key multistakeholder platforms in the health and sustainable development agenda and highlight any that overlap with the target health issue.
	National or local governance	To identify ways of working at national and local levels within the health and sustainable development agenda and highlight any that overlap with the target health issue.

In December 2023, during WCDP in Australia, a hybrid workshop (3 hours) with representatives from all Global Alliance member organisations was convened to discuss the draft results and the functionality of the Global Alliance. Representatives of WHO, CIPRB, RLSSA, RNLI, TGI and UNICEF later participated in a panel discussion during WCDP, which was transcribed to provide further data.

### Analysis

Our study used both deductive and inductive approaches to address our aims. Data collection was guided by our framework to identify potential intersections initially through document review and key interview interviews and coded deductively in line with the domains of our framework using Microsoft Word. Data focused on opportunities for integration of drowning prevention within UN Agency agendas were extracted from document review and key informant interviews. Themes were developed inductively and synthesised into the approach presented in table 2.

**Table 2:** Leveraging drowning prevention intersections across UN agencies using the Position, Add, Reach, Reframe approach

Approach	Description	WHO	IMO	FAO	UNDP	UNICEF
Position	Leverage governance pathways including global institutions, multistakeholder platforms and national governance to create opportunities for advocacy for drowning prevention, especially for global and country-level priority setting.	Position the need to address drowning prevention in high-level global and regional heath led agendas.	Position drowning prevention in context of national government Ministries of Maritime Safety and Transport.	Position drowning prevention in context of rural development, and riverine and coastal community development and well-being.	Position drowning prevention in context of civic leadership, climate and disaster resilience and UN priorities post SDGs.	Position drowning prevention in child and adolescent health, early childhood development, education, youth development and climate resilience, disaster risk reduction, health in emergencies, urban planning, and WASH agendas.
Add	Adjust workable solutions and capabilities (capacities, guidelines, and resources) to increase their relevance and impact on the health issue.	Add drowning prevention more specifically within high-level advocacy and guidelines for universal health coverage, social determinants of health, planetary health and climate health agendas.	Add drowning prevention considerations adjacent to safety at sea or fishing safety advocacy, guidelines and programmes.	Add drowning prevention to indicators for fisherfolk and fishing communities’ surveillance systems, and weather warning and safety systems.	Add drowning prevention to indicators for national and regional level reporting.	Add drowning prevention interventions, where feasible, in all community-facing health, education and development programmes.
Reach	Partner to extend the reach of health issue interventions into the affected communities where partners work or influence.	Provides potential to reach into affected communities through seeding demonstration projects, and programmes, status reports, indicators advocating national health reporting.	Reach into domestic ferry safety and potentially refugee safety in transit leveraging IMO capabilities for convening on maritime safety.	Reach riverine and coastal populations where burden of drowning in everyday life is identifiable.	Reach into plans and actions for Small Island Developing States and Least Developed Countries and Rural and less economically developed populations.	Reach populations with identifiable burden of drowning through country programmes.
Reframe	Describe shared problems, workable solutions, and interventions in a manner that reinforces their contribution to addressing the heath issue.	WHO has done much to reframe drowning prevention as an important public health issue and could expand reframing towards sustainable development agendas.	Reframe safety at sea and death at sea to link more clearly to drowning prevention.	Reframe fisher safety, deaths in fishing and fish farming as preventable drowning.	Reframe drowning prevention in context of community resilience and civic leadership.	Reframe drowning prevention interventions into agendas for child and youth development and child and adolescent health.

FAO, Food and Agriculture Organization; IMO, International Maritime Organization; SDGs, Sustainable Development Goals; UNDP, United Nations Development Programme; UNICEF, United Nations Children's Fund; WASH, Water, Sanitation and Hygiene.

All data were iteratively triangulated in Microsoft Word until the study reached thematic saturation to and integration within UN Agency agendas and to strengthen multisectoral action for drowning prevention. Member checking processes were followed. Draft results were provided to key informants, workshopped by the advisory group and then again returned to key informants for further checking prior to publication.

### Positionality and reflexivity

Our research team is multidisciplinary, multisectoral and multistakeholder. J-PS is a doctoral fellow, conducted all interviews and facilitated the workshop and panel discussion. J-PS, AR and SW are drowning prevention policy advocates and researchers. JJ and AR are LMIC injury prevention researchers with insights into the high drowning burden contexts. DM, and CL are WHO employees with responsibility for various areas of injury prevention, including drowning and act as secretariate to the Global Alliance. JAV is a UNICEF employee responsible for a range of health areas including injury prevention, including drowning prevention. JJ, DM, CL and JAV have expertise in policy analysis, public health and sustainable development agendas outside of drowning prevention. Positionality is further addressed in limitations, and our reflexivity statement is contained in online supplement 2.

### Patient and public involvement

There was no public/patient involvement in this study. Participants did not receive any compensation.

## Results

Our results are presented in line with the study aims.

### Framework for identifying intersections in health and sustainable development agendas

We developed our framework for identifying intersections in health and sustainable development agendas drawing on the key domains and factors identified through our analysis (table 1). The framework offers a systematic approach to identifying intersections in health and sustainable development agendas and proposes three domains based on the potential for (1) shared understandings of problems and solutions, (2) shared capacities, guidelines and resources and (3) shared governance pathways. The framework contains 10 factors including shared problems, commonalities in affected groups, workable solutions, core capabilities and strategic alignments, noting that some factors overlap.

#### Theoretical basis for the framework

Four bodies of research informed the development of the framework. First, Shiffman and Smith introduced the importance of issue characteristics, credible indicators, issue severity, effectiveness of interventions and political contexts like governance in the context of global health networks.^23^ These concepts have been expanded in our framework to reinforce the importance of affected groups in domain 1.^20^

Second, we reviewed approaches to mapping links between SDGs,^14 15^ which cross-reference overlapping SDG indicators.^24^ Nilsson *et al*^15^ introduced trade-offs and cobenefits in assessing intersections across SDGs and applied key factors such as geographical context, resource capabilities, time horizons and governance. This approach was tested but found challenging as drowning prevention is yet to develop an agreed set of indicators that could be easily mapped to the SDG framework in detail.

Third, we reviewed research mapping health or development topics across the SDGs. For example, in assessing where health sits across all SDGs, Nunes *et al*^16^ introduced a more thematic review using concepts like synergies and sectors specific to each SDG.

Finally, the literature reinforced concepts including typology for governance roles for health in multisectoral collaboration—leading, supporting and minimal^19^ or reviewing intersections based on interventions either being reinforcing, enabling or constraining, counteracting^15^ or based on interests, institutions and ideas.^25^

### Applying the framework to map drowning prevention within UN Agendas

Table 3 shows the application of our framework to mapping intersections for drowning prevention in the agendas of the UN Agency members of the Global Alliance. Data from 27 documents and reports, and UN Agency websites were reviewed and combined with data from our previous research.^9 10^

**Table 3:** Intersections for drowning prevention in UN Agency members of the Global Alliance for Drowning Prevention

Domain	Factor	WHO	IMO	FAO	UNDP	UNICEF
Identifying and building on shared understandings of problems and solutions	Shared problems	Addressing drowning is a key cause of injury mortality and morbidity.	Safety of seafarers, those working on ships and/or in fishing industry.	Occupational safety and health of fishers.	UNDP objectives are too diverse to identify specific direct links to mortality (see below)	Children and adolescent injury and mortality (drowning).
	Affected groups	WHO is dedicated to the well-being of all people and guided by science, leads and champions global efforts to give everyone, everywhere an equal chance to live a healthy life.	IMO support for affected groups includes: Seafarers (workforce) Passengers exposed to drowning or other accidents Refugees and migrants using water transportation in transit	FAO support for affected groups includes: Concerned with safety and health of all fishers and fish farmers in particular small-scale fisheries Most in coastal, marine, inland, river and delta fisheries	UNDP focus includes climate adaptation and building climate resilience in: Small Island Developing States and Least Developed Countries Rural and less economically developed populations	UNICEF supports governments to ensure the health and well-being of children and adolescents, including services for early childhood, disability, non-communicable diseases, environmental health and injuries.
	Workable solutions	WHO has published Global Reports and implementation guides on drowning, including recommending a framework of 10 actions to prevent drowning and providing technical guidelines for key interventions.	IMO applies: Mandatory instruments and model regulations (over 50) regulating aspects of shipping, including Convention on Safety of Life at Sea, Life Saving Appliances Code Technical co-operation and sea safety training	FAO solutions include: Improved design of fishing vessels (demonstrations, trainings) Develop and implement safety guidelines, codes and furnish policy assistance Provide technical assistance, training programmes Access to insurance and social protection International cooperation (IMO, ILO and WHO) on fishing safety	There is some evidence of UNDP involvement in local-level solutions to build community-based disaster resilience such as swimming or basic rescue and opportunities involve leveraging capacity for partnerships and inclusive multisectoral governance.	UNICEF approach includes: Advocacy and public awareness Safe places, barriers, daycare Safe journeys, safe boating, shipping and ferry regulations Safety skills—swim survival and basic rescue skills
Identifying and building on shared capacities, guidelines and resources	Core capabilities including: System Capacities Standards and guidelines Resources	WHO strengths are: Institutional power to convene actors and raise high-level awareness Potential for linking drowning prevention to a range of other health led agendas Publishing reports and guidelines Provides an informal secretariat for leadership for downing prevention	IMO capabilities include: Convening member states Developing codes of practice and regulations Alignment in constituencies including ministries for shipping, transport, agriculture, rural development Regional cooperation with organisations that regulate shipping that is, ASEAN, EU, South Pacific Community	In 1965 FAO was given the specific mandate to be the leading intergovernmental body in the field of fisheries. FAO capabilities include inter alia: policy and technical assistance to its 195 Members (including 194 countries and the European Union) in support of sustainable and safe fisheries and aquaculture activities across the value chain.	UNDP capabilities include: Poverty eradication Health Governance Financing Service delivery	UNICEF capabilities include: Advocacy Community engagement and behaviour change programmes Data, research, evaluation Public and private partnerships to accelerate SDGs and child rights
Identifying and building on shared governance pathways	Strategic alignment including: Global institutions Global strategies and plans Multistakeholder platforms National or local governance	WHO alignment includes: Direct advocacy and resolutions at the UN General Assembly and World Health Assembly. Positioning of drowning prevention within the division of universal health coverage and healthier populations provides for further positioning into other key health global multistakeholder platforms and plans. Governance and stakeholder arrangements for the Global Alliance	IMO strategic directions which offer promise include: Improve (safety) implementation Respond to climate change Engage in ocean governance Enhance global facilitation and security of international trade Address the human element	FAO strategic directions that offer promise include: Social dimensions including strengthening rural institutions and services, social protection, gender equality, decent rural employment, fisherfolk rights Inclusive rural transformation and revitalisation of rural areas ensuring equal participation of and benefits to poor, vulnerable and marginalised groups Economic transformation, social protections, fair work for small and medium fish farmer communities	UNDP signature solutions that offer promise include: Poverty—social protection, future of work through a systems lens, Health—reducing inequalities, Governance—Civic engagement, local governance, sustaining peace Localising SDGs—youth empowerment, Climate and disaster resilience	UNICEF strategic directions that offer promise include its aims to ensure every child, including adolescents: Survives and thrives Learns and acquires skills for the future Is protected from violence, exploitation, abuse, neglect and harmful practices Has access to safe and equitable WASH services and supplies and lives in a safe and sustainable climate and environment Has access to inclusive social protection and lives free from poverty

ASEAN, Association of South-East Asian Nations; EU, European Union; FAO, Food and Agriculture Organization; ILO, International Labour Organisation; IMO, International Maritime Organization; SDGs, Sustainable Development Goals; UNDP, United Nations Development Programme; UNICEF, United Nations Childrens Fund; WASH, Water, Sanitation and Hygiene.

WHO addresses drowning as a key cause of injury mortality and morbidity, which aligns with WHO’s dedication to the well-being of all people. This gives WHO great potential for policy reach into many affected groups, primarily but not solely through the health sector. The WHO recommended 10 actions (solutions) to prevent drowning and their implementation guidelines for key interventions are influential in guiding policy decisions.^4 26^

Drowning fits within the UNICEF objective to address mortality for children and adolescents, who account for more than 50% of the global drowning burden.^27 28^ UNICEF addresses key affected groups for drowning through extensive support for the health and well-being of children and adolescents. UNICEF aims to influence policy-making in child rights and supports many services designed to address early childhood care, disability, non-communicable diseases, environmental health and injuries which may also present opportunities for integration of drowning prevention policy.

A key component of IMO’s mandate is the safety of seafarers, those working on ships and/or in fishing industry.^29^ Although outside of its mandate, the IMO has increased efforts to assist Member States to address domestic passenger vessel safety through model regulations,^30^ technical cooperation and capacity-building activities.^31^ IMO is also being called to consider the safety of refugees and migrants in transit in waters, who are classified as passengers, meaning IMO may provide intersections in affected communities for drowning prevention.

FAO assists all States inter alia to ensure fishing activities allow for safe, healthy and fair working conditions and environment.^32 33^ Most accidents and injuries happen in small-scale fisheries, which account for 90% of total capture fisheries employment. FAO works with the safety of coastal, inland, river and delta small-scale fishing communities, who are affected by vulnerabilities and exposures to drowning. Improved sea safety, which includes occupational health and safety, may be best achieved through improved design of fishing vessels, the development and implementation of safety guidelines, safety codes, training programmes and coherent and integrated national strategies with the participation of the fishers themselves.^34 35^

UNDP objectives are diverse and often address underlying causes of poverty and other social determinants which can be linked to drowning prevention.^36^ UNDP’s policy influence, specifically its investment in community-level governance and resilience, including across cross-cutting themes like gender equality,^37^ has potential for integration of drowning prevention solutions. UNDP often works to address upstream economic and social determinants of health, which may also reduce vulnerabilities and exposure to drowning. Mandates for ending poverty, advancing equity and education may also help to reduce community-level drowning vulnerabilities and exposures.^2^ UNDP’s focus on Small Island Developing States and Least Developed Countries works to accelerate climate adaptation, both contexts are considered priorities for drowning prevention.^9^

### Applying the Position, Add, Reach, Reframe approach to multisectoral action for drowning prevention

We identified a range of opportunities for integrating drowning prevention to the UN Agency agendas and arranged them into categories, which were refined during the steering committee workshop. These categories, Position, Add, Reach and Reframe, were further refined and present an approach to identifying opportunities for the integration of drowning prevention within other health and sustainable development agendas. The approach is described below and outlined in table 2.

#### Position: create high-level positioning for drowning prevention

Positioning drowning prevention within relevant health and sustainable development agendas is a strategic priority.^9^ Key informants indicated that UN Agencies often contribute to these agendas and engage in high-level advocacy that might benefit drowning prevention. UN Agency influence on global and regional governance, participation in multistakeholder platforms and national priority setting could be further leveraged to advance drowning prevention objectives.

Potential examples for expanded positioning of drowning prevention include WHO, UNICEF and UNDP’s involvement in climate and health agendas, UNICEF and WHO’s involvement in child and adolescent health and development agendas, UNDP and UNICEF’s promotion of civic leadership, and all UN Agency member contributions to the prioritisation of disaster risk reduction.

Suggested tactics for positioning drowning prevention include advocacy via side meetings, the inclusion of drowning prevention as a topic on meeting agendas and ensuring that drowning is explicitly considered in guidance, research and as part of key global initiatives (eg, the climate change agenda).

#### Add: layers of drowning prevention to existing solutions and capabilities

Key informants suggested that some workable solutions and capabilities could be adjusted to increase their relevance and impact on drowning prevention. This approach might provide cobenefits by extending existing solutions or adding policy advocacy for drowning prevention within UN Agency workstreams or subsequent programming.

A suggested example was the UN focus on data for decision-making. Adding drowning to existing surveillance systems is minor step but would increase visibility and collect data to inform solutions. Further, it leverages existing capabilities and offers opportunities to implement change through research, technology, governance and capacity-building.

Another example cited was improved housing, which might reduce a resident’s exposure to water sources, even without the need for partnership with the drowning prevention field. The benefit could be greatly strengthened if such housing initiatives added design measures like barriers and fencing to water to explicitly reduce drowning exposure for children.

#### Reach: extend reach for drowning prevention to affected communities

Key informants reinforced the challenges of scaling known evidence-informed interventions to reach the most vulnerable populations and suggested UN Agencies could help address this implementation gap. An example provided was working with UNDP as a conduit to building partnerships that reach and focus on the high rates of drowning in Small Island Developing States.

Key informants also stressed indirect benefits where programmes that incidentally reduce vulnerabilities, exposures or build resilience to drowning, may be drawn on to extend reach into affected communities. Programmes addressing social determinants, improved working and living conditions, improved infrastructure or school attendance can all reduce drowning vulnerabilities and exposures and present opportunities to extend reach.

#### Reframe: shared problems and workable solutions as contributing to drowning prevention

Key informants identified existing solutions delivered by UN Agencies that likely contribute to drowning prevention where linkages could be reframed, and the links made more explicit.

Suggested examples were ‘safety at sea’ and ‘maritime safety’ which both contain approaches designed to prevent drowning but are rarely described as drowning prevention. Other examples cited related to UNICEF policy areas where some workstreams could be reframed to make their linkages with drowning prevention more obvious.^38^

### Key themes for advancing the Global Alliance and Global Strategy

The following themes emerged in response to the question of how best to advance drowning prevention strategically, and with the aim of informing the development and implementation of the Global Alliance and Global Strategy.

#### Ensuring national and community-level impact

Key informants recognised that momentum for drowning prevention has been generated through UNGA and WHA resolutions, the establishment of the Global Alliance and the intention to develop a strategy, but that initiating community-level impact should be a high priority.

*The UN resolution was secured and the World Health Assembly resolution, it was a big celebration, clearly. But to me it was the start, not the end. And I had to keep reminding my colleagues and myself that until something changes at a community level, nothing has changed.* (Interview—non-state actor)

There were strong views that the Global Alliance and Global Strategy should provide a bridge from high-level advocacy to country level to influence community outcomes, meaning that success should be measured on the extent of traction for drowning prevention achieved at national and subnational levels.

*I think it needs to be grounded in country level actions and accountability. But I think giving guidance and setting minimum standards for national strategies will be important as well.* (Interview—UN Agency)

Key informants reinforced the need to strengthen accountability at country level using clear governmental policy targets, and indicators for investment in and implementation of measures. These targets and indicators could be aligned to either resolution or a purpose-built accountability framework developed in the context of the Global Strategy.

#### Generating incentives for the integration of drowning prevention

UN Agency representatives voiced the need for tools to facilitate and build internal resourcing while cautioning that the tools needed to be ‘more carrot than stick.’ Many suggested establishing cobenefits and exploring opportunities to add value to existing workstreams.

*We could find a way to create organizational incentives and just to make the people who want to work on drowning prevention have a little bit more leverage when they go to their bosses and say, this Alliance has identified (insert) as having a key role and this fits with our existing work on this.* (Interview—UN Agency)

#### Strengthening multisectoral action and involvement

Key informants reinforced that many sectors are involved in drowning prevention, particularly at the government level. An important strength of the UN Agencies is their convening power and ability to coordinate their reach to extend into other ministries, stakeholders and sectors, which are critical to drowning prevention at country level.

*It will vary from country to country. From Bangladesh perspective, I see that this is Ministry of Women and Children Affairs, then Ministry of Health and Family Welfare, Ministry of Social Welfare, Ministry of Education. Minister of Local Government, to some extent, Department of Fire service and Civil defence to some extent.* (Interview—non-state actor)

Key informants reinforced the need to build on existing mandates, explaining that some UN Agencies face pressure from constituents, often Member States or other UN Agencies, to use their capability or convening power to address issues considered adjacent to their core mandates. This factor might create opportunities to leverage existing mandates into a focus on drowning prevention.

*The fatality statistics for ferry accidents, especially in Asia and Africa, were so abominable that the Member States decided we needed to do something about it. But since we cannot mandatorily regulate domestic affairs, what we have done is we have model regulations for domestic ferry safety.* (Interview—UN Agency)

A commonly expressed view was that the Global Alliance should focus on building connections and integration within sustainable development agendas and their associated programmes including the SDG targets.

*I think there'll be core interventions and core focus areas. But really, the point of the Global Alliance is to tackle drowning prevention from all sides and looking at it from the perspective of environmentally, economically, and socially and everything.* (Interview—UN Agency)

Many thought that ensuring drowning prevention is referenced in future iterations of the SDGs and/or the UN Agency strategies where relevant should be a long-term objective. For example, daycare— a key drowning prevention intervention, links conceptually and in practice to SDG targets for access to quality early childhood development, care and preprimary education.

Key informants described searching key policy publications for drowning and most often failing to find any reference. This point reinforced the importance of advocacy, generation of evidence and case studies focused on the objective of broadened policy-maker awareness of drowning prevention.

*I searched (a major global report) to see if they included anything from various injury perspectives. They are clearly talking about heat related injuries and how those impact. There’s very little mention of water, drowning, probably rightly so, but maybe not.* (Interview—non-state actor)

#### Increasing investment

Funding and investment were a recurring theme. There are challenges for financing drowning prevention, especially in the context of extending known interventions to scale, and advocacy and capacity-building aimed at building national government responsibility for policies and programmes.

*(Global Alliance should be) aligned with messaging and increasing global awareness on the important interventions and maybe even potentially pushing governments for investment. You know, hopefully we can utilize one voice and get some momentum and government investment.* (Interview—non-state actor)

Others cited opportunities to address funding for research and suggested the development of tools for resourcing, grant making, fundraising and participation in joint calls for funding.

#### Building an effective operating model

Key informants repeated the belief that the Global Alliance should achieve more than the sum of the activities of members. Some members—particularly non-state actors—wanted to ensure that the Global Alliance undertook some monitoring and evaluation of its efforts to confirm that it was adding value.

*We had a discussion that we must be better than the status quo. So, if you start to see drowning prevention waning because of any work that’s done under the notion of an alliance, then we should just pack it up and go home and leave the sector to get on with the job because we're doing well to start with.* (Panel—non-state actor)

There is also a view that the Global Alliance should be explicit about the resources needed to sustain activity and operate the secretariat. In this regard, it was reinforced that WHO had made an intentional decision to start small.

*We initially wanted to start with a relatively small number of entities because it’s a complicated process, because we are dealing with a complicated problem. We need to wrap our minds about how we can coordinate and collaborate in a more efficient manner.* (Panel—UN Agency)

There is a tension between efficiency, resources and impact, meaning consideration should be given to the resource requirement to sustain the administration of Global Alliance activities, membership and secretariat including any future use of working groups.

*I'm a bit nervous about this Alliance, to be frank, because I've seen my colleagues, who work with large alliances, large partnerships, which have become sort of very large, not very nimble entities and are difficult to work with.* (Interview—UN Agency)

#### Expanding global engagement

There was some conjecture about the preliminary membership of the Global Alliance among delegates at the WCDP in December 2023, specifically about which non-state entities are included initially. The workshop and panel discussed the matter and agreed that end user or community benefit should guide future stakeholder engagement models.

*So, we've just got to focus not on the politics of missing out, but to focus on how this has an impact on harnessing the energy of the community, this community. This is fundamentally about enabling the agenda to continue to accelerate for the benefit of those most at risk of drowning.* (Panel—non-state actor)

There are UN Agencies with potential to influence drowning prevention which have not yet joined the Global Alliance. Examples include the World Meteorological Organization, the International Labour Organization, the United Nations Office for Disaster Risk Reduction, United Nations Educational, Scientific and Cultural Organization and the International Organization for Migration.

*One of the most useful things would be that we now have a substantial convening power spread across, now, five UN agencies. But I'm quite optimistic that within a brief period, we can have some other UN agencies that we really wanted to have join this alliance to begin with, to come with us.* (Panel—UN Agency)

Key informants suggested that research mapping of drowning prevention within these UN agencies may identify opportunities to include drowning prevention in disaster risk and flood prevention, early childhood, primary and secondary education, and migration and refugee agendas. Several suggested this research should seek to initiate partnerships with multilateral development banks including the World Bank and the Asian Development Bank.

#### Identifying collective views on advancing a Global Strategy

A workshop agreement was that the Global Strategy should be focused on advancing drowning prevention rather than specifically the Global Alliance itself, meaning it should have utility for stakeholders beyond Global Alliance members. This requires the Global Strategy to be multistakeholder and multisectoral in its approach, having relevance and providing tools for a wide range of actors.

Another workshop agreement was that the Global Strategy should be set up with established targets/indicators and resource requirements as well as a monitoring and evaluation framework.

### Developing a logic model

**Figure 2:**
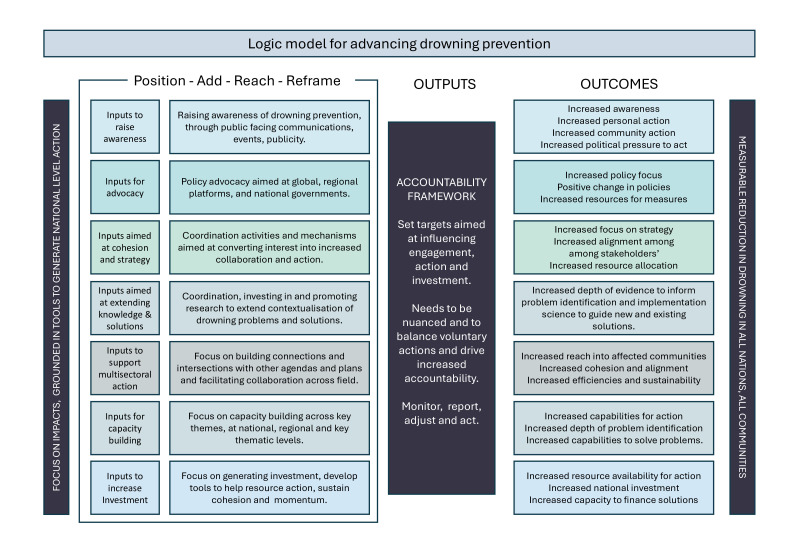
Logic model for advancing drowning prevention.

Identified priority inputs, activities, outputs and outcomes for advancing drowning prevention, the Global Alliance and Global Strategy are organised into the logic model (figure 2). These included raising public awareness, external advocacy targeting governments, promoting national drowning prevention agendas through policy targets, voluntary responsibilities and highlighting case studies, and inputs aimed at fostering cohesion and sustaining action.

## Discussion

Our study responds to increasing UN Agency engagement in drowning prevention, as outlined in the WHA resolution^6^ and builds on WHO commitments to establish a Global Alliance and develop a Global Strategy for drowning prevention. Our multimethod qualitative approach uses an advisory group committee, document review, key informant interviews, interagency workshop and conference panel and has resulted in several codeveloped outcomes relevant to our aims. Our discussion is presented in three sections:

### The proposed framework in the context of multisectoral action

Our framework for identifying intersections in health and sustainable development agendas drew on theories published in research on the linkages between SDGs,^14 15^ integrating health across the SDGs^16^ and literature on governance in multisectoral collaborations.^17^,^19^

We reviewed approaches designed to explain the emergence and effectiveness of global health networks which use the domains of problem definition, positioning, coalition-building and governance in analysis.^20 21^ While applied to understand prioritisation of single issues including early childhood development,^39^ violence against children^40^ and maternal heath,^23^ these factors may also be applied to explain how two seemingly separate issues or agendas can combine, or how one network can partner with another.

Approaches like Shiffman and Smith, Nunes, and the mapping of indicators in SDGs offer valuable insights, including that partnership is more art than science and that a ‘one-size-fits-all’ approach has limitations when implementing multisectoral frameworks. Instead, an evolving and amalgamated framework that draws on the strengths of existing models to provide a pragmatic approach for real-world application is very much needed.

While partnerships are a necessary tool for multisectoral action, frameworks for operationalising them in an integrated manner are sometimes lacking.^41^ Our framework seeks to identify actionable strategies and create policy coherence in the potential partnerships that address multisectoral action for drowning prevention.

The use of concepts like shared mortality, affected communities and workable solutions provided the basis for categorising opportunities for drowning prevention and flowed well into the Position, Add, Reach and Reframe approach adopted in our analysis. Both techniques (the framework and approach) could assist in the identification of actionable strategies in the planning of multisectoral action for drowning prevention beyond those identified in this study, including at national and community level.

### Establishing a Global Alliance

Our study considers potential priorities to ensure the Global Alliance is established in a manner that will sustain the current momentum for drowning prevention. In our logic model, we reinforce the importance of building internal cohesion, strengthening external advocacy, raising awareness, capacity-building and increasing research contextualising problems and solutions. All of our key informants agreed that having and measuring impact from global to country level is essential to the future effectiveness of the Global Alliance. However, we identified three challenges that must be addressed in the early development processes.

First, the operating model of the Global Alliance needs further and likely ongoing consideration. While the first steps are indicative of a need to start small and focus on impact not administration, there is healthy tension between resourcing global level activities and interests, and the imperative of impacting outcomes at community level. Many recognise the widespread resource challenges facing those seeking action on drowning prevention, especially the need for increased investment and funding directed towards national and community level—where it is needed most. By choosing an alliance model described as semiformal, as opposed to a more structured legal entity-based partnership, efforts to capture, focus and mobilise the energy and resources of Global Alliance members and other groups will be critical to success.

Second, accountability is a repeated and complex theme. Accountability at Global Alliance level will include internal WHO reporting lines and reporting at WHA level. There are also layered expectations and performance pressure from across stakeholders currently external to the Global Alliance membership. The development of an accountability framework directed at all stakeholders, including Member States, is a critical component of any future Global Strategy.

Finally, drowning prevention contexts are diverse making it challenging to reflect all voices in global leadership and governance, but it is imperative that those most affected are central to all decision-making. Inclusive practices, in both the Global Alliance and the Global Strategy, must be developed and implemented to mitigate the risks of entrenching inequitable relationships and unbalanced distributions of power and resources that can often be experienced in other global health partnerships.^42^

### Building on priorities for a Global Strategy

Our study reinforced the need for multistakeholder and multisectoral approaches to the Global Strategy, including elements and tools to guide implementation like targets, indicators, resource requirements, recommended actions and the development of an evaluation framework.

The audience for the Global Strategy is not disputed. It is intended to influence the whole field, including those not yet engaged in drowning prevention. The overarching aim to achieve measurable reductions in drowning too, has universal support. Where more critical thought is needed is the question of function. We recommend that the Global Strategy should focus on three areas.

First, strengthening the capacity of the drowning prevention field. The Global Strategy should establish concrete plans to grow the capabilities of the field for effective action by guiding a research agenda, communicating clear priorities, resourcing capacity-building and addressing gaps in best practices through implementation research, guideline development and technical exchanges.

Second, expanding the reach of drowning prevention to affected communities. The Global Strategy should aim to expand reach through high-level political advocacy, engagement with health and sustainable development agendas and raising public awareness to drive community action and put political pressure on decision-makers to act.

Finally, ensuring that the Global Strategy drives tangible change aimed at community impact. The Global Strategy should establish a multisectoral framework for action with clear indicators, incentives to develop and deliver evidence-informed measures and a planned cycle of implementation, review and adjustment so that momentum can be tracked

### Strengths and limitations

Our study has several strengths worth highlighting.

First, the methods used (key informant interviews, document analysis, interagency workshop and panel discussion), and the insights and results, have all impacted the maturation of ideas and consensus for the Global Alliance and Global Strategy among steering committee members. Shared learning is essential to collaboration, but that learning must now extend beyond current Global Alliance members.

Second, the piloted framework for identifying the intersections in health and sustainable development agendas is novel and has been applied here to map intersections for drowning prevention in UN Agency members of the Global Alliance. The framework should also be used to map opportunities for drowning prevention in other sustainable development agendas. With further refinement the framework could be applied in other health and development policy areas to scope opportunities for multisectoral action or partnership between two or more health and sustainable development agendas.

Our study is not without limitations. Membership of the Global Alliance defined participation in our research team and advisory group, which can be considered to lack contextual representation from Africa, Central and South America, and Small Island Nations. Our research team and advisory group does not represent national or community-level actors, nor does it adequately reflect representation from all countries and contexts with high drowning burden (except JJ and AR). J-PS, SW, DM, CL and JAV, while HIC based, have long-term experience and responsibilities for policy and research implementation in low and middle income countries (LMICs). This may have affected our methodological approach and results.

Our key informant interviews and interagency workshop participation were limited to Global Alliance steering committee members. Going beyond the steering committee to include UN agencies not currently engaged in the Global Alliance and to include other drowning prevention stakeholders may have provided further insights and strengthened our results.

Likewise, our mapping of intersections was restricted to current UN Agency members of the Global Alliance. Mapping other potential UN Agency agendas for further examples of shared problems, overlaps in affected communities, and workable solutions may add value in future studies.

## Conclusion

Our study reinforces the importance of working with UN Agencies to advance high-level positioning of drowning prevention and ensure national and community-level impact. It supports the need for increased investment and the develpment of incentives for the integration of drowning prevention within other agendas including those focused on child and adolescent health, climate health and resilience, disaster risk reduction, maritime safety, domestic water transport and broader development.

Our framework, approach and logic model can be used to advance the drowning prevention fields capabilities for effective and evidence-informed action with an emphasis on building capacity and best practices; expanding influence and reach into affected communities through high-level political advocacy; and building Member State commitments and accountability to national and community-level action.

We conclude that the Global Alliance must establish an operating model to provide a bridge between high-level accountabilities and community-level impacts. The Global Alliance must seek to expand global engagement to address the expectations of stakeholders, manage growth in membership, drive further engagement of wider groups, and prioritise the inclusion of those most affected in decision-making.

Finally, plans to develop a Global Strategy for drowning revention will evolve, and the strategy development process itself presents further opportunities to identify and build shared understandings of problems and solutions, foster inclusive leadership and global governance and expand evidence-informed measures to reach deeper into communities affected by drowning.

## Supplementary material

10.1136/bmjgh-2024-016125 online supplemental file 1

10.1136/bmjgh-2024-016125 online supplemental file 2

## Data Availability

Data are available on reasonable request.
