# Supplementary material for: A framework for identifying opportunities for multisectoral action for drowning prevention in health and sustainable development agendas: a multimethod approach
Source: BMJ Glob Health. 2024 Aug 22;9(8):e016125. doi: 10.1136/bmjgh-2024-016125 (PMC11404292; doi:10.1136/bmjgh-2024-016125)
Supplement: online supplemental file 1 [file bmjgh-9-8-s001.pdf]

Supplement 1

Key informant Interview Questions

Title:
A framework for identifying intersections for drowning prevention in health and sustainable development agendas: mixed methods approach.

Ethics:
HC No: 200929

Aim:
This study aims to 1) build a framework for identifying intersections between health and sustainable development agendas, 2) apply the framework to map links for drowning prevention in United Nations agency agendas, and 3) inform the development and implementation of the Global Alliance for Drowning Prevention and Global Strategy.

Interview questions guided by author derived framework.

Interviewers note:

Ensure participant has no concerns regarding participation and understand the consent process.

Ensure signed consent form has been received.

Proceed with interview if participant confirms to the following eligibility criteria.

- An actor engaged in global drowning prevention leadership, advocacy, research, and practice.
- A national level actor considered to have or have knowledge of factors that influenced global or regional advocacy efforts.
- An actor considered to be central to the emergence and sustained political prioritisation of global drowning prevention.
- An actor from an international organisation, international non-government organisations, non-government organisations, academic institutions, government, or donor.

QUESTIONS:
ESTABLISHING SHARED UNDERSTANDING OF PROBLEM AND SOLUTIONS
(Problems, Affected groups, workable solution)
Identifying shared problems and solutions for MSA for drowning prevention in mapped agenda.

Questions:

- Are there any areas of (insert) where mortality or morbidity overlaps with drowning. What is the context, who is affected and what solutions does (insert) bring to the problem.
- When considering drowning prevention (prompt – adjusted WHO actions), are there solutions that would likely resonate most within your agenda?
- Are you able to identify any populations or communities or contexts where (insert) works that may have an elevated drowning burden?

CREATING SHARED FRAMING, CONCEPTS AND POSITIONING
(Framing, Concepts, Themes)
Identifying areas of existing or potential shared positioning for MSA for drowning prevention in mapped agenda.

Questions:

- If I attended major conference for (insert), what are the key concepts that a drowning prevention person would most need to understand to engage its audience effectively? Where are the commonalities?
- What are the major themes within (insert) strategic agenda, that present opportunities for drowning prevention?
- If you had to explain the link between drowning prevention and (insert) how would what key words would you use?

ENGAGING SHARED STAKEHOLDERS AND SECTORS
(Stakeholders, sectors, donors)
Identifying shared or potential for shared constituencies for MSA for drowning prevention in mapped agenda.

Questions:

- Who are the key stakeholders represented within (insert) agendas?
- Who are the key sectors represented within (insert) agendas?
- Do any of them also contribute to drowning prevention (upstream or downstream)?
- Who are the contributors to advancing (insert) agenda, what is the basis of their support?

LINKING SHARED TOOLS
Identifying tools (capacity, standards, resources) for MSA for drowning prevention in (insert).

- Can you identify an example in (insert) where multisectoral approach has been successively implemented in partnership with other UN agencies? What do you think are the lessons relevant to drowning prevention.
- What are the key capacities with (insert) that should be leveraged for MSA for drowning prevention? (prompt – resources, systems, people, guidelines, relationships).
- Thinking about shared solutions mentioned earlier, what are the systems and guidelines (policies, agreements) that should be explored for mutual benefit?

BUILDING ON SHARED GOVERNANCE PATHWAYS
(Institutions, strategies, or plans, multisectoral platforms, national/local, leadership)
Identifying pathways for governance for MSA for drowning prevention in mapped agenda.

- When working with (insert) what the key instruments that a drowning prevention strategy must align with or contribute to?
- Can you identify global alliances that (insert agenda) works with a governance / partnership level, what is the impact and how do they work best.
