# Supplementary material for: A framework for identifying opportunities for multisectoral action for drowning prevention in health and sustainable development agendas: a multimethod approach
Source: BMJ Glob Health. 2024 Aug 22;9(8):e016125. doi: 10.1136/bmjgh-2024-016125 (PMC11404292; doi:10.1136/bmjgh-2024-016125)
Supplement: online supplemental file 2 [file bmjgh-9-8-s002.pdf]

Reflexivity Statement

1. **How does this study address local research and policy priorities?**

This study is global level policy and is not intended to directly address local research and policy priorities.

2. **How were local researchers involved in study design?**

This study was situated at global level. JJ and AR are LMIC injury prevention researchers with insights into the high drowning burden contexts.

3. **How has funding been used to support the local research team?**

There was no local team and there was no funding specific to this research project.

4. **How are research staff who conducted data collection acknowledged?**

All contributors were offered co-authorship. Those who contributed but did not seek authorship are acknowledged in the acknowledgements section. None of those who opted not to contribute as coauthors are from LMICs.

5. **Do all members of the research partnership have access to study data?**

All members of the research team and advisory group (Global Alliance Steering Committee) have access to data.

6. **How was data used to develop analytical skills within the partnership?**

The research team and advisory group (Global Alliance Steering Committee) participated in meetings, workshops, and had access to data, interim results and analysis. All contributed throughout, and therefore had the opportunity to develop analytical skills.

7. **How have research partners collaborated in interpreting study data?**

The research team and advisory group (Global Alliance Steering Committee) participated in meetings, workshops, and had access to data, interim results and analysis. All contributed to data interpretation.

8. **How were research partners supported to develop writing skills?**

The research team writing this article is a doctoral student (J-PS), supported by his doctoral supervisor (JJ), and senior academics and policy specialists.

9. **How will research products be shared to address local needs?**

This study will be published as open access. The results will be shared with attendees of the World Conference on Drowning Prevention 2023. Approximately 25% of attendees were from LMICs.

10. **How is the leadership, contribution and ownership of this work by LMIC researchers recognised within the authorship?**

Authors JJ and AR are part of the senior authorship team in developing this manuscript, and their contribution has been recognised as last authors (JJ), and in the respectively.

11. **How have early career researchers across the partnership been included within the authorship team?**

J-PS is a doctoral student and is lead author on this project.

12. **How has gender balance been addressed within the authorship?**

Three authors are female (CL, JV and JJ (last author)) and four authors male (J-PS (lead author), DM, AR, and SW).

13. **How has the project contributed to training of LMIC researchers?**

The project was not conducted in a LMIC setting. It did not have a training component. However, the context and implications of the projects are most relevant to LMIC, high drowning burden countries. The authorship team is primarily composed of senior researchers. All the authors based in low- and middle-income countries are senior researchers.

14. How has the project contributed to improvements in local infrastructure?

It is hard to define “local” in the context of this project. This project has not directly contributed to improvements in local infrastructure.

15. What safeguarding procedures were used to protect local study participants and researchers?

Safeguarding procedures are not relevant/applicable to the context of this study. The study was approved by the Human Research Ethics Advisory Panel D: Biomedical at The University of New South Wales (Reference: HC220687).
